# Comparison of urine heavy metals in exclusive menthol and non-menthol cigarette users by race/ethnicity: The 2015–2016 National Health and Nutrition Examination Survey Special Sample

**DOI:** 10.18332/tpc/167389

**Published:** 2023-06-28

**Authors:** Wenxue Lin

**Affiliations:** 1 Department of Epidemiology and Biostatistics, College of Public Health, Temple University, Philadelphia, United States

**Keywords:** NHANES, menthol, cigarette, biomarker, race/ethnicity, heavy metals

## Abstract

**INTRODUCTION:**

The objective of this study was to investigate the differences in urine concentrations of heavy metals (uranium, cadmium, and lead) between exclusive menthol and non-menthol cigarette smokers across three racial/ethnic groups using data from the National Health and Nutrition Examination Survey (NHANES) 2015–2016 Special Sample.

**METHODS:**

Data from NHANES 2015–2016 Special Sample were analyzed to assess the association between menthol smoking and heavy metal biomarkers in urine across three racial/ethnic groups (N=351), including Non-Hispanic White (NHW), Non-Hispanic Black (NHB), and Hispanic/Other (HISPO). Multivariable linear regression models were used to estimate adjusted geometric means (GMs) and ratio of GMs (menthol/non-menthol smokers) (RGMs) for urine biomarkers of heavy metals between menthol and non-menthol smokers by race/ethnicity.

**RESULTS:**

Among the 351 eligible participants, 34.4% (n=121) were NHW, 33.6% (n=118) were NHB, and 32.0% (n=112) were HISPO exclusive cigarette smokers. The analysis revealed significantly higher concentrations of urine uranium in NHB menthol smokers compared to NHB non-menthol smokers (RGMs=1.3; 95% CI: 1.0–1.6; p=0.04). NHW menthol smokers appeared to have higher levels of urine uranium than non-menthol smokers, but the difference was not statistically significant (9.0 vs 6.3; RGMs=1.4; 95% CI: 1.0–2.2; p=0.08). There were no significant differences in urine metals (cadmium and lead) by menthol status among NHW, NHB, or HISPO cigarette smokers (p>0.05).

**CONCLUSIONS:**

The research findings regarding the higher levels of urine uranium among Non-Hispanic Black (NHB) menthol cigarette smokers raise questions about the claims suggesting that additives in cigarettes do not contribute to increased toxicity.

## INTRODUCTION

The aggressive marketing of menthol cigarettes by tobacco companies has had a disproportionate impact on African Americans^1^, contributing to 1.5 million new smokers and >150000 smoking-related deaths among Blacks from 1980 to 2018 ^2^. Additionally, racial/ethnic differences in nicotine metabolism have been well-established, with non-Hispanic Black (NHB) smokers exhibiting higher serum cotinine levels despite smoking fewer cigarettes per day compared to non-Hispanic White (NHW) smokers^3-6^. However, few studies have investigated the impact of menthol on urinary biomarkers of tobacco exposure on exclusive cigarette smokers among NHW, NHB, and Hispanic/Other (HISPO).

While a recent study using data from the National Health and Nutrition Examination Survey (NHANES) Special Sample found that menthol and non-menthol cigarettes deliver similar levels of harmful and potentially harmful constituents^7^, the impact of menthol on urinary biomarkers of heavy metal across different racial/ethnic groups has not been fully explored. Therefore, the purpose of current study is to expand upon previous research^7^ by examining differences in urinary heavy metal concentrations between menthol and non-menthol exclusive cigarette smokers across three racial/ethnic groups using data from the NHANES 2015–2016 Special Sample. By investigating these differences, the aim is to provide insight into the potential health risks associated with menthol cigarette smoking among different racial/ethnic groups and to contribute to ongoing efforts to address tobacco-related health disparities.

## METHODS

The National Health and Nutrition Examination Survey (NHANES) was used for the current cross-sectional study. The NHANES 2015–2016 Special Sample collected urine samples from participants aged ≥18 years, including non-smokers and oversampled adult smokers who used at least 100 cigarettes in their lifetime and smoked daily. The study focused on exclusive cigarette smokers, and those who used other tobacco or nicotine products within the last five days were excluded. After excluding non-smokers and observations with missing data, there were 351 exclusive cigarette smokers, with 34.4% (121/351) NHW, 33.6% (118/351) NHB, and 32.0% (112/351) HISPO exclusive cigarette smokers. Out of the 351 exclusive cigarette smokers included in the study, 162 (46%) were identified as menthol cigarette users, while the remaining 189 (54%) were categorized as non-menthol cigarette users. Sociodemographic variables were collected through NHANES surveys, including age at screening, gender (male, female), race/ethnicity (Non-Hispanic White, Non-Hispanic Black, Hispanic/Other), education level (less than high school, high school or higher), body mass index (BMI, kg/m^2^), and ratio of family income to poverty. Information on smoking status, cigarettes smoked per day, cigarette stick length, and cigarette menthol indicator was obtained from cigarette use and recent tobacco use surveys. Urinary biomarkers, such as heavy metal (uranium, cadmium, and lead) concentrations were corrected for dilution by creatinine and are reported as ng per g of creatinine^8^.

Multivariable linear regression models were used to analyze the association between menthol and urine concentrations of heavy metals by race/ethnicity. The results are expressed as adjusted geometric means (GMs) and ratio of GMs (RGMs) between smokers of menthol and non-menthol cigarette, by race/ethnicity. Given the non-normal distribution of urine biomarkers, natural log-transformation was used to satisfy the normality assumptions^8^. The geometric mean (GM) was employed instead of the arithmetic mean to analyze the transformed biomarker values, ensuring more accurate and meaningful comparisons within the study. The ratios of the geometric means and their 95% CIs were obtained by exponentiation from the linear regression models on log-transformed biomarker levels^9^. For urine biomarkers, in addition to log transformation, creatinine adjustment was used to minimize the effects of variation of analyte concentration in urine ^8^. SAS SURVEY procedures were used for all statistical analyses ^10^.

## RESULTS

Table 1 displays the adjusted geometric means of urinary heavy metal concentrations between menthol and non-menthol cigarette smokers, by race/ethnicity. Out of the total 351 participants in the study who exclusively smoked cigarettes, 34.4% (121/351) were Non-Hispanic White (NHW), 33.6% (118/351) were Non-Hispanic Black (NHB), and 32.0% (112/351) were Hispanic/Other (HISPO) exclusive cigarette smokers. Among NHB, the ratios of the adjusted geometric means comparing menthol with non-menthol cigarette smokers were 1.3 (95% CI: 1.0–1.6; p=0.04) for uranium, 1.2 (95% CI: 0.9–1.5; p>0.1) for cadmium, and 1.0 (95% CI: 0.8–1.4; p>0.5) for lead (Table 1). NHW appeared to have a higher level of urine uranium among menthol smokers than non-menthol smokers, but the difference was not statistically significant (9.0 vs 6.3; RGMs=1.4; 95% CI: 1.0–2.2; p=0.08). There was no other significant difference in urine metals (cadmium and lead) by menthol status among NHW, NHB or HISPO cigarette smokers (p>0.05) (Table 1). Further details regarding the calculations and analysis are given in the Supplementary file.

**Table 1:** Adjusted geometric means of heavy metal concentrations^a^ between menthol and non-menthol cigarette smokers by race/ethnicity, a cross-sectional study of the National Health and Nutrition Examination Survey Special Sample, United States, 2015–2016

Biomarker*	*Menthol GM (95% CI)*	*Non-menthol GM (95% CI)*	*RGMs (95 % CI )*	*p*
**Non-Hispanic White** (N=121)				
Uranium	9.0 (4.9–16.8)	6.3 (4.3–9.0)	1.4 (1.0–2.2)	0.08
Cadmium	283.7 (217.7–369.7)	377.3 (339.9–418.8)	0.8 (0.6–1.0)	0.06
Lead	377.8 (284.2–502.2)	452.6 (376.1–544.8)	0.8 (0.7–1.1)	0.12
**Non-Hispanic Black** (N=118)				
Uranium	6.3 (5.0–7.9)	5.0 (4.2–6.0)	1.3 (1.0–1.6)	**0.04**
Cadmium	354.6 (306.6–410.1)	304.2 (242.5–381.6)	1.2 (0.9–1.5)	0.20
Lead	349.7 (312.3–391.6)	339.6 (265.0–435.2)	1.0 (0.8–1.4)	0.84
**Hispanic/Other** (N=112)				
Uranium	6.5 (4.6–9.1)	7.0 (5.0–9.7)	0.9 (0.6–1.4)	0.70
Cadmium	330.6 (243.6–448.6)	261.9 (223.2–307.2)	1.3 (1.0–1.6)	0.07
Lead	423.1 (307.8–581.8)	432.3 (348.3–536.6)	1.0 (0.7–1.4)	0.90

* Adjusted for gender, education level, cigarette stick length, age, BMI, ratio of family income poverty, and cigarettes per day. GM: geometric mean. RGMs: ratio of geometric means (menthol/non-menthol).

a Heavy metal (uranium, cadmium, and lead) concentrations were corrected for dilution by urinary creatinine and are reported as ng per g of creatinine.

## DISCUSSION

NHB menthol cigarette users had significantly higher urine uranium concentrations than NHB non-menthol cigarette users. These findings are concerning given the known carcinogenic properties of uranium and the negative health impacts of menthol-flavored cigarettes on Black smokers. Uranium and thorium are radioactive carcinogens found in smoke from burning cigarettes^11^. Deposits of radioactive uranium may contribute to localized radiation exposures in lungs. Moreover, when combined with other non-radioactive carcinogens from smoke, uranium can have a synergistic effect that increases the risk of developing cancer^11^. Mint species are recognized for their capability to accumulate metals from the soil, including uranium^12^. African Americans make up <13% of the overall United States population, but the use of menthol cigarettes has been linked to increased likelihood of initiation, lower quitting rates, delayed cessation, disparities in smoking-related health outcomes, and death in Black smokers^2^. The increased urine uranium concentrations observed from menthol NHB cigarette smokers may help to explain the negative health impacts of menthol-flavored cigarettes on Black smokers.

Reducing nicotine content in cigarettes has been proposed as a potential strategy to reduce smoking-related health risks. A clinical trial study found benefits of reduced nicotine content cigarettes^13^. However, menthol-flavored cigarettes may negatively impact the treatment effects of reduced nicotine content cigarettes^14,15^. For instance, Denlinger-Apte et al.^14^ found that menthol significantly diminished the treatment effect of Very Low Nicotine Content (VLNC) cigarettes compared to non-menthol VLNC. Similar results were found in another trial where menthol VLNC smokers with low socioeconomic status experienced smaller degree of reduction in cotinine compared to non-menthol VLNC smokers^15^. These findings suggest that strategies to reduce nicotine content in cigarettes should take into account the potential negative impact of menthol.

### Limitations

The generalizability of the study findings is limited by the inclusion of cigarette smokers only. Further, the study used United States NHANES 2015–2016 Special Sample and thus current findings are not generalizable to smokers who reside in other less developed countries, since the health impact of smoking can vary depending on the tobacco control policies and the level of industrialization in a country^16^. In addition, knowledge and beliefs regarding harm from different tobacco products^17,18^ might be another important factor to guide the Food and Drug Administration (FDA) nicotine reduction policy, given the close relationship between risk perceptions and smoking behavior^19^. Furthermore, it is important to note that the study does not consider other environmental factors and individual-specific factors that could potentially influence the levels of uranium in urine. An additional limitation of the study is the relatively small sample size, as only data from the NHANES 2015–2016 special sample were analyzed.

## CONCLUSIONS

The research findings of this study, regarding the higher levels of urine uranium among Non-Hispanic Black menthol cigarette smokers, raise questions about the claims suggesting that additives in cigarettes do not contribute to increased toxicity.

## Data Availability

The NHANES data are publicly available at https://www.cdc.gov/nchs/nhanes/index.htm
