# Supplementary material for: Comparison of urine heavy metals in exclusive menthol and non-menthol cigarette users by race/ethnicity: The 2015–2016 National Health and Nutrition Examination Survey Special Sample
Source: Tob Prev Cessat. 2023 Jun 28;9:22. doi: 10.18332/tpc/167389 (PMC10302213; doi:10.18332/tpc/167389)
Supplement: Supplementary file 1 [file TPC-9-22-s1.pdf]

Table S1. Calculations, formulas, and SAS keywords for analysis*

Metal	Calculations and formulas
Uranium (ng/g creatinine)	log((URXUCD/URXUCR)*100000)
Cadmium(ng/g creatinine)	log((URXUPB/URXUCR)*100000)
Lead(ng/g creatinine)	log((URXUUR/URXUCR)*100000)
	SAS Keyword
Uranium	URXUUR
Cadmium	URXUCD
Lead	URXUPB
	PROC SURVEYREG
	STRATA
	CLUSTER
	WEIGHT
	DOMAIN
	LSMEANS

*For detailed calculations, formulas, and statistical analysis methods, please access the NHANES tutorial at the following website: https://wwwn.cdc.gov/nchs/nhanes/tutorials/default.aspx The tutorial provides comprehensive guidance and instructions on various aspects of data analysis using NHANES data, including but not limited to sample design, weighting, variance estimation, reliability of estimates, and software tips.

Table S2. Univariate Association between tobacco consumption (number of cigarettes smoked per day) and concentrations of metals (ng/g creatinine)

Independent Variable	Dependent Variable	p-value
Cigarettes smoked per day	Uranium (ng/g creatinine)	0.2189
Cigarettes smoked per day	Cadmium (ng/g creatinine)	**0.0039**
Cigarettes smoked per day	Lead (ng/g creatinine)	**0.0031**

© 2023 Lin W.
